# Implications of Prenatal Exposure to Endocrine-Disrupting Chemicals in Offspring Development: A Narrative Review

**DOI:** 10.3390/nu16111556

**Published:** 2024-05-21

**Authors:** Juan M. Toledano, Maria Puche-Juarez, Jorge Moreno-Fernandez, Patricia Gonzalez-Palacios, Ana Rivas, Julio J. Ochoa, Javier Diaz-Castro

**Affiliations:** 1 Department of Physiology, Faculty of Pharmacy, Campus Universitario de Cartuja, University of Granada, 18071 Granada, Spain; jmtoledano@ugr.es (J.M.T.); jjoh@ugr.es (J.J.O.); javierdc@ugr.es (J.D.-C.); 2 Institute of Nutrition and Food Technology “José Mataix Verdú”, University of Granada, 18071 Granada, Spain; pgonzapa@ugr.es; 3 Nutrition and Food Sciences Ph.D. Program, University of Granada, 18071 Granada, Spain; 4 Instituto de Investigación Biosanitaria (IBS), 18016 Granada, Spain; amrivas@ugr.es; 5 Department of Nutrition and Food Science, University of Granada, 18071 Granada, Spain

**Keywords:** endocrine-disrupting chemical, newborn, non communicable diseases, fetal programming, cardiometabolic diseases, neurodevelopment

## Abstract

During the last decades, endocrine-disrupting chemicals (EDCs) have attracted the attention of the scientific community, as a result of a deepened understanding of their effects on human health. These compounds, which can reach populations through the food chain and a number of daily life products, are known to modify the activity of the endocrine system. Regarding vulnerable groups like pregnant mothers, the potential damage they can cause increases their importance, since it is the health of two lives that is at risk. EDCs can affect the gestation process, altering fetal development, and eventually inducing the appearance of many disorders in their childhood and/or adulthood. Because of this, several of these substances have been studied to clarify the influence of their prenatal exposure on the cognitive and psychomotor development of the newborn, together with the appearance of non-communicable diseases and other disorders. The most novel research on the subject has been gathered in this narrative review, with the aim of clarifying the current knowledge on the subject. EDCs have shown, through different studies involving both animal and human investigation, a detrimental effect on the development of children exposed to the during pregnancy, sometimes with sex-specific outcomes. However, some other studies have failed to find these associations, which highlights the need for deeper and more rigorous research, that will provide an even more solid foundation for the establishment of policies against the extended use of these chemicals.

## 1. Introduction

Different pathways of the endocrine system can be altered by a variety of natural and synthetic chemicals, modifying its activity, and consequently leading to negative health results for population. The compounds responsible for these effects are known as endocrine-disrupting chemicals (EDCs), and are included in several daily-life items, such as food-packaging, cosmetics, toys, flame retardants, or detergents, as well as in pesticides and other widely used chemicals [1]. EDCs affect the homeostasis of the endocrine system in many stages, which includes the alteration of the synthesis, liberation, binding, transport, metabolism and/or elimination of important hormones. These exogenous substances have a ubiquitous presence, and their detrimental implications on human health has made them an object of deep scientific research, showing the risk that these chemicals represent [2]. In fact, policies related to EDCs have been developed by several institutions with the aim of regulating these substances’ applications. These policies have increasingly augmented their strictness with the passage of time according to the insights provided by scientific community to avoid an excessive exposure to these potentially damaging molecules [3].

Among their wide range of effects, the damage to the reproductive system of both men and women is one of the most relevant abilities EDCs have demonstrated. Their detrimental effect appears even before conception, as they disrupt the fertility of both progenitors [4]. Nevertheless, their impact is more perceptible during gestation, due to the fact that it is one of the most sensitive life stages with regard to the environment. These chemicals interact with several hormones related to development and reproduction, representing an undoubted potential for endocrine disruptors to produce irreparable damage to both mothers and their offspring [1]. Pregnant women can come into contact with these substances through many ways, including the skin, the food chain, or even the respiratory tract, widely spreading to different organs and tissues. In this sense, several studies highlight that EDCs can be found in several human samples, including serum, urine, and even amniotic fluid and breast milk. In addition to this, some of them can reach the placenta and even accumulate there, undermining its formation and homeostatic functions [5]. This organ plays a key role during gestation, since it promotes fetal homeostasis by carrying out the exchange of substances (gasses, nutrients, and waits) and the transport of signaling molecules required for its proper growth, also acting as a physical barrier to protect it from environmental insults [3]. Nevertheless, the placenta is not utterly impenetrable, being permeable to some EDCs which can cross it and reach fetal circulation. The immaturity of the fetus and its high cell differentiation rate makes it especially vulnerable to external factors, so small modifications in protein and hormone activity would generate a severe impact [3]. This impact might be related to a higher risk of pregnancy complications during the gestation process, including preterm delivery, intrauterine growth restriction, gestational diabetes mellitus (GDM), and preeclampsia [6]. Of note, these pregnancy outcomes have increased their prevalence during the past years, as a result of an increase in population’s exposure rate to these disrupting chemicals [7].

Nevertheless, the potentially detrimental impact of EDCs is not just restricted to the pregnancy, since subtle modifications related to these substances might exert relevant effects on forming tissues, which would in turn change the developmental course of the new life. These gathered effects are known as “early programming”, and are considered a considerable risk factor for the appearance of non-communicable diseases (NCDs) later in life [8]. Barker’s “Developmental origins of health and disease hypothesis” (DOHaD) states that preconception, gestation, and the first two years of life have a major influence in the future health state [9]. Indeed, endocrine disruptors have been associated with adipogenesis, obesity, diabetes, and cardiometabolic problems, together with defects in cognitive/psychomotor development and behavior [10,11], since some of them are able to cross the fetal blood–brain barrier [12]. Additionally, certain chemicals have reported a potential association with malformations in the fetus, respiratory alterations, and even the eventual appearance of some cancers, especially breast, ovarian, and prostate cancer [13].

Although a number of negative effects have been observed, the certain damage these chemicals can cause during fetal and postnatal life have not been fully elucidated to date. The long half-life that many of these compounds have and the possibility of their accumulation in some tissues makes it important to consider not only their exposure during gestation, but also during the whole life course of the mother until pregnancy takes place. The current scientific literature even provides contradictory results in some cases, when it comes to the short- and long-term impact that EDCs would exert, and how it would affect offspring development and health, so further research is required to finally clarify this matter.

## 2. Materials and Methods

The bibliographic research was carried out from April 2023 to July 2023. Using the primary biomedical databases and sources—Medline (via PubMed), Elsevier, The Cochrane Library, and Dialnet—the search was narrowed down to the years 2018–2023. Only pertinent articles from recent publications that addressed the topic of this study (the developmental and programming impacts on offspring induced by prenatal exposure to EDCs) were accepted. Because English is the primary language used in science, only papers written in that language were included in the search. The used keywords were as follows: endocrine disruptor, phthalates, bisphenols, pesticides, polycyclic aromatic hydrocarbons, parabens, perfluorinated compounds, prenatal exposure, pregnancy, programming effects, obesity, adiposity, diabetes, early development. Terms from the medical subject heading (MSH) were utilized in those words that could lead to a misunderstanding in the browser. Moreover, the Boolean operators “AND”, “OR”, and “NOT” were used in conjunction with keywords to locate more pertinent articles. “AND” was used between each term to increase the sensitivity and specificity of the search. “OR” was used to link synonyms. In order to prevent browser confusion, “NOT” was not used frequently.

**Figure 1:**
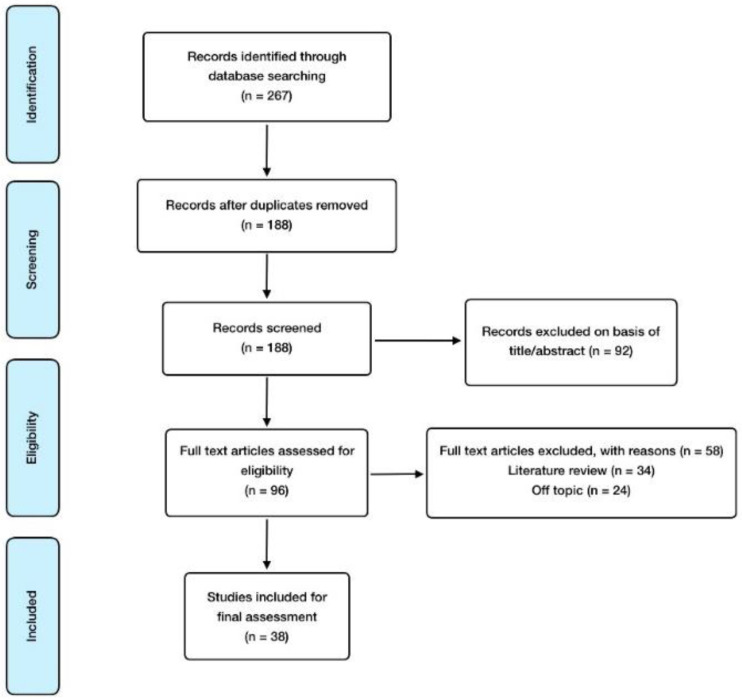
Manuscript selection flowchart.

With regard to inclusion criteria, these were the following: controlled trials, observational studies, animal model, in vitro studies, and meta-analysis; and English language, especially considering those articles involving alterations in the offspring derived from prenatal EDC exposure. As for exclusion criteria, these were the following: abstract absence and language other than English. The reference software used for article management, citation, and bibliography organization was EndNote. Finally, the search methodology and the article selection process developed is reflected in Figure 1.

## 3. Results and Discussion

### 3.1. Endocrine Disruptive Chemicals (EDCs)

According to the World Health Organization (WHO), an endocrine-disrupting chemical is “an exogenous substance or mixture that alters function(s) of the endocrine system and consequently causes adverse health effects in an intact organism, its progeny, or (sub)populations” [4]. This heterogeneous group of more than 800 chemicals produces an endocrine alterations by mimicking or antagonizing the metabolism of some hormones, including thyroid hormones, estrogens, and androgens [1]. Among the extensive variety of endocrine disruptors, this review focuses on the groups described in this section, as they show stronger evidence in the scientific literature regarding their effects on fetal development and offspring alterations [14].

Starting with the most studied group of EDCs, bisphenols are synthetic and industrial substances widely used in the production of epoxy resins and polycarbonate plastics. Polycarbonate plastics are frequently applied to elaborate food packaging, while epoxy resins are used in composite materials to coat metal objects like cans. As a consequence, diet is the most common source of exposure to them, together with other daily products containing bisphenols (e.g., medical devices, coating powders, and thermal printing papers) [15]. Their high solubility in organic solvents makes them accumulate more easily in the body’s lipid compartments [3]. The most used chemical of the group has always been bisphenol A (BPA), even though the current tendency is to try to exchange it for other allegedly less-damaging analogs, such as bisphenol S (BPS), bisphenol F (BPF), or bisphenol AF (BPAF). Nevertheless, their adverse effects have proved to be similar, and sometimes, more powerful than those caused by BPA [16]. BPA’s estrogenic activity is widely known, as it is able to successfully activate estrogenic receptors (ERs). Its main metabolite generated in the body, BPA monoglucuronide (BPA-G), does not exert this disrupting activity, even though it has been reported to cause pro-inflammatory effects [17].

Phthalate esters (PAEs), are synthetic esters of phthalic acid widely used to improve the characteristics of a number of plastics, including flexibility, durability, and softness, and are also applied as stabilizing agents. Therefore, they are utilized for the manufacturing of several products, such as food packaging, toys, personal care products, building materials, medical equipment, and textiles among others [3]. This makes them some of the most extended chemicals, with higher levels found in female subjects [18]. Interestingly, pthalathes have reported higher toxicity after their metabolization to monoesters [19]. Diethyl phthalate (DEP) and di(2-ethylhexyl) phthalate (DEHP) are the most common among these chemicals, with monoethyl phthalate (MEP) as the main metabolite of DEP, whereas DEHP is metabolized into several substances, such as mono(2-ethylhexyl) phthalate (MEHP), mono(2-ethyl-5-hydroxyhexyl) phthalate (MEHHP), mono(2-ethyl-5-carboxypentyl) phthalate (MECPP), and mono(2-ethyl-5-oxohexyl) phthalate (MEOH) [20]. However, there are other PAEs which have not been as thoroughly studied by the scientific community in terms of human effects, so they need to be further investigated to properly evaluate their short- and long-term toxicity. These include di-iso-nonyl phthalate (DINP), benzyl butyl phthalate (BBP), dimethyl phthalate (DMP), di-n-butyl phthalate (DBP), di-n-octyl phthalate (DNOP), mono-iso-butyl phthalate (MIBP), mono-(3-carboxypropyl) phthalate (MCPP), monobutyl phthalate (MBP), mono-benzyl phthalate (MBZP) mono-n-butylphthalate (MNBP), monocarboxy-isononly phthalate (MCNP), mono-(2-ethyl-5-oxohexyl) phthalate (MEOHP), and mono-carboxy-isooctyl phthalate (MCOP) [4,21].

Organochlorine pesticides (OCPs) are other relevant EDCs to consider, which are organic substances made of carbon, hydrogen, and chlorine atoms. They have been applied as pesticides for insects, fungus, and weed control, and represent 40% of all pesticides used around the world. This group of substances includes the widely banned (even though some countries still use it) dichlorodiphenyltrichloroethane (DDT) and its metabolites; as well as hexachlorobenzene, lindane, and dieldrin [22]. Their high affinity for organic solvents makes them accumulate easily in the adipose tissue, just like some other endocrine disruptors. Another negative characteristic of these persistent organic pollutants (POPs) is related to their capacity to be transmitted through the food chain, finally reaching human beings [22]. Additionally, many of them can cross the placenta and reach the fetus, representing a high risk for the development of the fetus [23]. Organophosphates (OPs) are another kind of pesticide, widely used as insecticides because of their ability to interfere with acetylcholine neurotransmission. Not being as persistent within the human organism, scientific research on organophosphates concerning prenatal exposure-derived alterations is scarce [24]. Pyrethroids are another type of chemical increasingly utilized to substitute other more toxic pesticides, even though they may also exert adverse effects for human health [25].

Polycyclic aromatic hydrocarbons (PAHs) are a group of chemicals that includes polychlorinated biphenyls (PCBs), which are synthetic substances utilized in electrical equipment and building materials. Although they have also been widely forbidden by many governments worldwide, there still exists an important source of exposure through contact with previously manufactured products containing them, as they have been frequently applied in mixtures including a variety of PCBs. They have been reported to have an environmental persistence, which leads to long-term implications for human beings [26]. On the other hand, polybrominated diphenyl ethers (PBDEs) are also persistent PAHs with a similar chemical structure, used as flame retardants in several products, such as plastics, textiles, paints, electrical equipment, and foams. The less brominated of these substances show greater toxicity since they are more prone to accumulate within living organisms due to their high affinity for lipids [27].

Another group of EDCs is either named as perfluorinated compounds (PFCs) or perfluorinated alkylated substances (PFASs), having been commonly applied in the manufacturing of non-stick cookware, waterproof clothing, anti-fouling paints, and firefighting foams. Perfluorooctanoic acid (PFOA) and perfluorooctane sulfonate (PFOS) are the most addressed members of this group of substances when it comes to scientific research, even though others may be more environmentally persistent [28]. However, they are also environmentally persistent, making prenatal exposure to them particularly concerning [29]. With regard to p-hydroxybenzoic acid (PHB) esters, also known as parabens, their antimicrobial properties make them suitable to be used as preservative agents in personal care products [30], though they also show endocrine disruptive implications, especially in children [31]. Nonylphenol ethoxylates (NPEs) are another group of compounds which are extensively applied as detergents, emulsifiers, and dispersants. Their decomposition product nonylphenol (NP) is widely spread, and as a result of its high hydrophobicity and low degradation rate, it can persist in waters, solids, and organisms, thus entering the food chain and reaching human beings through contaminated drinking water, food, breast milk, food packaging, and other products like those related to personal care [32]. As for phytoestrogens, they are plant compounds which exert weak estrogenic activity, being present in a number of plants, particularly soy and their derivates, but also grains, beans, vegetables, and fruits. There are three main types of phytoestrogens: isoflavones like genistein, glycitein, and daidzein; coumestans, like coumestrol; and lignans [33].

Human beings undergo exposure to a wide range of synthetic chemicals daily, through dietary components, physical factors, and psychosocial stressors, which may, in turn, trigger a variety of biological responses. All of them are included within the term “exposome”, representing a complex relationship between individuals and their environment which makes it difficult to point out an individual exposure as the single cause of a concrete health outcome. However, they may be considered as possible mediators of health alterations, knowledge of which would be of great importance to fully understand the potential causes involved [34]. In this sense, the articles showing the relationships between prenatal EDC exposure and offspring development are contained in Table 1, including the addressed EDC, the study design, and the principal findings observed in them.

### 3.2. Prenatal EDCs Exposure and Changes in Adipose Metabolism and Obesity

EDCs are known to disrupt important metabolic signaling pathways, such as PPARs, estrogen receptors (ERs), and thyroid hormone receptors (THRs), as well as to operate as obesogens, which are compounds that can change energy balance by encouraging adipogenesis and fat buildup, even during the gestation process. This obesogenic effect might be associated with two detrimental consequences. On the one hand, it may influence the outcome of the gestation process, as the presence of obesity, diabetes, and cardiometabolic conditions during pregnancy is widely associated with the appearance of maternal and fetal complications [73,74], and exposure to EDCs is one of the risk factors for the appearance of such comorbidities. On the other hand, it may also be related to obesity development in the offspring [75].

On the first subject, the association between BPA, PAEs and obesity in reproductive-aged women and women during pregnancy has been studied. Regarding BPA, a cross-sectional study reported that exposure was related to body mass index (BMI), also finding a slight association with waist circumference [76]. Another similar study supported these findings by discovering a correlation between BPA urinary concentrations and both BMI and waist circumference [77]. As for phthalates, higher urinary MEHP, MEHHP, MEOHP, and MECPP concentrations were related to increased odds for central obesity compared with lower exposures [78]. On the contrary, another study assessing MEHHP, MEOHP, and MNBP levels failed to show an association with any anthropometric and metabolic parameter [79]. According to this, it is not possible to draw definitive conclusions on this potential relationship.

As for newborns, early life exposure to EDCs could possibly affect obesity epigenetic programming, due to their ability to bind nuclear receptors such as PPAR-γ, which is a key adipogenesis regulator that controls the expression of several metabolic genes during cell differentiation. These effects are accompanied by modified methylation of both PPAR-γ and their target genes [80]. It has been observed that the way PPAR-γ genes are expressed during the gestation process is definitive to determine if mesenchymal stem cells will differentiate into osteocytes or adipocytes, which has a major influence on body fat accumulation [81]. Bisphenols are the main EDCs linked to obesity that are currently under extensive research [4]. In vitro studies have demonstrated that primary adipose progenitor cells are susceptible to endocrine disruption by BPA, which impairs proliferation and differentiation in the fetus [35]. Mice models have reported that prenatal BPS exposure can increase susceptibility to adipogenesis induced by a high-fat diet in F1 generation, through an upregulation of PPAR-γ activity and consequently, an overexpression of their target genes [36]. Both BPA and BPS have also been related to oxidative stress and augmented adipocyte differentiation, leading to obesity [82,83]. Long-term BPA exposure, to a wide range of doses, has also been seen to trigger a dose-dependent increase in both body and liver weight in male descendants, whereas females showed a body weight reduction [84]. Similar experiments in rats resulted in offspring fatty acid accumulation, which led to hepatic steatosis [85]. Furthermore, maternal BPA exposure was also demonstrated to alter the expression of several key genes related to adipogenesis regulation in female offspring, including PPAR, sterol regulatory element binding protein 1c (SREBP-1c), stearoyl-CoA desaturase 1 (SCD-1), and CCAAT-enhancer-binding protein α (C/EBPα) [86]. These findings are supported by evidence provided from extensive recent research linking intrauterine exposure to childhood obesity [87]. The development of adiposity at later ages (5 and 7 years of age) was found to be influenced by prenatal BPA exposure in a New York longitudinal cohort, with relationships between prenatal BPA exposure and greater BMI, body fat percentage, and waist circumference [88]. Other studies also found sex differences, with a positive association between prenatal BPA exposure and increased waist-to-hip ratio, waist circumference, and subscapular fold in girls, but not in boys [37]. Another study from China found the same relationships, as well as an increased risk of central adiposity, only among girls [38]. Despite this fact, there is still controversy since studies have not demonstrated a strong link between fetal BPA exposure and obesity at 10 years of age [39].

PFCs have also been assessed when it comes to their possible influence on pregnant mothers and offspring adiposity. In humans, maternal serum PFASs concentrations have been associated with increased risk of low birth weight, which is one of the predictors of increased adiposity in adulthood [89]. However, a recent study found out that PFASs levels were related to lower adiposity at 8 years of age, and only when separated by type, PFOA (one of the main types of PFASs) was associated with higher waist circumference and higher IL-1β levels, suggesting greater inflammation, although results are not very consistent [40]. The association of PFASs with adiposity is also highly dependent on other factors such as maternal obesity or parental ethnicity [41], so exposure to them may be a more modifiable factor in the epigenetics of adiposity than these other ones. Additionally, there may also be an association based on baby’s gender, as was demonstrated in a study where negative associations were found for boys, but positive ones were reported among girls, based on various aspects related to obesity such as BMI, waist circumference, and body fat mass in 7-year-old children [42]. Supporting the link between PFASs and obesity, a meta-analysis of several cohort studies has found an increase in overweight children and an augmentation in BMI Z score per PFOA concentrations measured in maternal blood [43].

**Figure 2:**
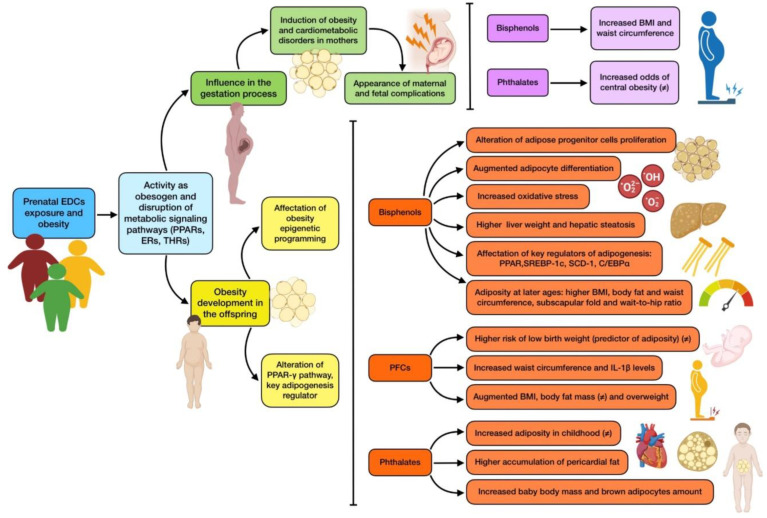
Summary of the main associations found between EDCs prenatal exposure and obesity. The existence of controversy on the effect due to mixed results is indicated with (≠).

The results of phthalate-based studies are not as reliable as those based on other EDCs, with less consistent associations between urinary concentrations during pregnancy and adiposity in childhood [44] and at 8 years of age [90]. PAEs and pericardial fat seem to be associated when the different phthalate types are taken into consideration, even though there is a lack of association with overall adiposity [39]. However, there have been some correlations between prenatal phthalate exposure and low adiposity at birth, but high adiposity at 5 [45] and 6 years of age [46]. As for gender differences, associations for PAEs and child adipocytes seem to be stronger among girls, as some studies have reported a correlation between prenatal exposure and BMI Z score [47,87]. Nevertheless, other research identified associations between phthalate exposure and adiposity unrelated to sex [91]. The type of phthalates that induce adipose effects differ from one study to the other, due to their differential estrogenic/antiandrogenic properties and different PPAR-γ-related activity [87]. The most solid findings can be seen in animal models, where it is shown that prenatal exposure to these EDCs is associated with increased baby body mass [92] and brown adipocytes amount, which would eventually result in an obesity issue [93]. Figure 2 summarizes the main effects on adipose metabolism and obesity induced by prenatal EDCs exposure.

### 3.3. Prenatal ECDs Exposure and Changes in Glucose Metabolism and Diabetes

The scientific literature shows how EDCs can augment the possibility of a thrifty phenotype. The thrifty phenotype hypothesis proposes that epidemiological associations between poor fetal/infant growth and the following development of type 2 diabetes and metabolic syndrome are the result of poor nutrition in early life, which induces permanent changes in the glucose–insulin metabolism and raises adult cardiometabolic risk [11]. Within this context, a great deal of animal and epidemiological studies show growing evidence for the role of prenatal ECD exposure (especially to bisphenols) in the development of glucose metabolism disorders. An increase in carbohydrate metabolism, together with a reduction in physical activity has been found in mice developmentally exposed to BPA, although these effects were only observed among female offspring [94]. Nevertheless, the diabetogenic effect of prenatal BPA exposure seems to be consistent in the scientific literature. For instance, a study demonstrated that BPA administration from day 9 to 16 of gestation produced glucose intolerance and insulin resistance, and dampened pancreatic ß-cell activity in male descendants after 6 months of age, while neither female nor younger male mice displayed these consequences [95]. These consequences were also exhibited later in life in the mothers themselves, due to the fact that, even though metabolic alterations disappeared after parturition, similarly to many cases of GDM, this remission was just temporary and manifestations reappeared months later. Non-pregnant treated mice experienced no effects, which points out that both BPA exposure and pregnancy are necessary conditions to generate this altered phenotype. Interestingly, the diabetogenic effects were also accompanied by a rise in fat accumulation and body weight [95,96].

Another study reported insulin resistance at 21 weeks of age in the offspring of rats orally exposed to BPA during pregnancy [97]. It is noteworthy that glucose metabolism alterations associated with BPA have been found not only in F1 generations, but also in F2, exemplifying a transgenerational influence, at least in rodent models [98,99]. Other alterations have also been reported regarding glucose metabolism, like hastened differentiation and delayed maturation of islets in the fetal pancreas of mice from mothers fed with a BPA diet between embryonic day 7.5 to 18.5. An increase in glucagon liberation was also reported in this study, indicating a possible imbalance in the α/β-cell ratio in pancreatic islets [100]. A relevant study exposed Wistar rats to BPA oral doses during gestation and lactation, detecting abnormal hepatic DNA methylation which preceded the appearance of insulin resistance in male offspring, between the 3rd and the 21st postnatal week [101]. On the other hand, in utero exposure to other EDCs, including pesticides and dioxins, has shown to confer increased risk of developing type 2 diabetes in mice [102]. Finally, a human cross-sectional study reported a correlation between BPA urinary concentrations and both fasting insulin and homeostatic model assessment of insulin resistance (HOMA-IR index) [77]. Taking all these findings into consideration, it can be suggested that bisphenols may have a relevant contribution to the development of metabolic disorders associated with glucose homeostasis, these effects being dose, time, and offspring sex dependent. As can be noticed, the vast majority of studies regarding glucose metabolism and EDCs are focused on bisphenols (especially BPA), so there is an important gap in the scientific knowledge to be filled.

### 3.4. Prenatal EDCs Exposure and Cardiovascular Health

Heart affectation has been a subject of deep study regarding prenatal EDC exposure. There is a great body of evidence provided from studies using zebrafish as an animal model. One of them exposed embryos to different concentrations of DBP, resulting in a high rate of cardiac malformation and looping, pericardial edema, and heart function alteration. To further understand the underlying mechanisms of this cardiotoxicity, the expression of key cardiac transcription factors such as NKX2.5 and TBX5 was measured, finding that it has been significantly reduced by DBP exposure in a dose-dependent way [48]. A similar experience was found with BBP, another frequently used phthalate that also reported cardiac defects, including malformations, reduced heartbeat, and increased distance between the sinus venosus and bulbus arteriosus. The expression of cardiac transcription factors was also dose dependent, and downregulated by the EDC [103]. A recent study evaluated the potential heart toxicity produced by six phthalates, including DMP, DEP, DBP, DEHP, DNOP, and BBP, revealing that all six PAEs induced abnormalities in zebrafish embryos, such as decreased heartbeat and pericardial edema [49].

Evidence on cardiovascular health has also been provided by rodent-based research. For example, administration of combined doses of BPA and PFOS to rats for 19 days during gestation induced morphological changes in the fetal heart. These included an approximate 20% increase in the interventricular septal (IVS) thickness, together with a rise in total collagen and dynamin-related protein 1 (DRP1) levels, whereas cell number did not significantly change. The combination of these two EDCs showed a synergistic effect on IVS thickness. These effects were investigated in vitro, showing an augmentation in cardiomyocytes size and collagen content [50]. Phthalates’ cardiotoxicity has also been assessed in mice exposed to prenatal doses of DEHP within 8.5–18.5 days of pregnancy, reporting increased apoptosis in cardiac cells, slowed myocardial sarcomere development, reduced heart weight, and cardiac septal alteration. The neuregulin 1 (NRG1) dependent regulation of the ErbB signaling pathway, which involves the activity of epidermal growth factor (EGF) and epidermal growth factor receptor (EGFR) (deeply associated with proliferation and differentiation) has been proposed to be the mechanism involved in DEHP myocardial cytotoxicity [51]. DEHP has also been associated with decreased expression of angiotensin II receptors in the adrenal gland, which consequently leads to reduced levels of circulating aldosterone. This could have implications on water and electrolyte balance, together with systemic blood pressure control. In fact, in utero exposure to this phthalate from gestational day 14 in rats resulted in systolic and diastolic arterial pressure reduction at postnatal day 200 [104]. Another EDC which has been studied because of its heart-related toxicity is nonylphenol, suggesting mitochondrial damage as a possible explanation of the alterations produced by this substance [105].

**Figure 3:**
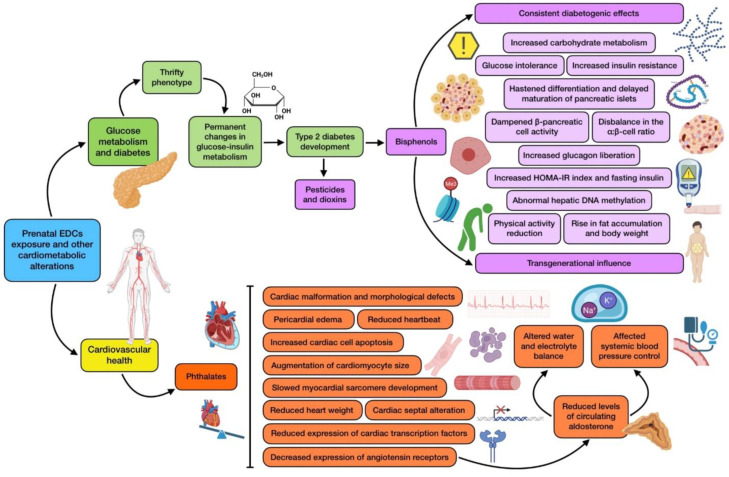
Summary of the main associations found between EDCs prenatal exposure and cardiometabolic alterations (diabetes and cardiovascular health).

On the other hand, a human cohort involving 1300 children has also been studied to explore the relationship between parental EDC exposure and congenital heart defects. Maternal occupational exposure to PAEs seemed to be related to perimembranous ventricular septal defect (PmVSD), secundum atrial septal defect (s-ASD), patent ductus arteriosus (PDA), and pulmonary valve stenosis (PS). As for paternal occupational PAEs exposure, it seemed to be associated with both PmVSD and PS [106]. These results support, through human-based research, the consistent evidence provided by animal studies on the risk that exposure to EDCs (and especially phthalates) represents for the development of cardiovascular disorders. The most relevant effects produced by prenatal EDC exposure on both diabetes and cardiometabolic health are shown in Figure 3.

### 3.5. Prenatal EDCs Exposure and Cognitive Development

The development of the human nervous system is a complex process that begins around the third week of pregnancy with the formation and closure of the neural tube [107]. During the prenatal (and particularly, in the third trimester of gestation) and early postnatal period, the central nervous system (CNS) is very sensitive to environmental stressors, as it is immersed in a critical development phase, hence the potential role of EDCs as risk factors for neurodevelopmental disturbances [108]. Prenatal exposure to these chemicals may affect fetal brain development through the disruption of two hormonal pathways, related to both thyroid and sex hormones. These alterations might have permanent and/or lifelong repercussions for children, such as cognitive and behavioral dysfunction, autism spectrum disorder (ASD), or attention deficit disorder [10].

With regard to BPA, this EDC can bind to ERs and may affect thyroid and gonadal hormone signaling, thus affecting regular brain development and subsequent behavioral patterns [109]. Rat models have shown that exposure to BPA doses within the range of human acceptable daily intake can alter the sexual differentiation of the neural structures and affect fetus behavior [110]. Although this substance is being replaced by alternatives like BPS or BPF, it is suggested that they may exert comparable effects, as has been reported by some animal studies [111]. A recent study found that prenatal BPS and BPA exposure affects cognitive development in 2-year-old children, measured by the Bayley scales [52]. There is evidence of a sex-dependent association, with males being more frequently related to prenatal exposure and ASD development than girls [53]. In general, most of the human-based studies assessing PBA exposure and child behavior have reported a detrimental association. When sex-specific outcomes were addressed, boys were more prone to show increased behavioral effects [112,113], while few studies have reported these effects among girls [114].

As for phthalates, they may impact infant health mainly by oxidative stress induction. Elevated oxidative stress indicators during pregnancy are correlated with urine phthalate metabolites, as well as with neurodevelopmental issues [115]. On the other hand, PAEs interaction with thyroid hormones during gestation also has negative consequences on fetus neurodevelopment, which may be noticeable later in infancy [116]. The most recent evidence indicates a direct relationship with ASD and hyperactivity [54], although this association seems to be gender-specific, being greater in females [117]. A systematic review has also highlighted how outcomes are different for girls and boys depending on the type of phthalate exposure during pregnancy. In this sense, adverse behavioral and cognitive outcomes proved to be more common among boys when exposed to low-molecular-weight phthalates, while in females, this assumption was true if exposed to high-molecular-weight ones [118]. Other research, however, has shown no connection between prenatal exposure to these chemicals and overall cognitive development [55]. For instance, a study examining 6-year-old infants reported that negative effects on attentional performance and intelligence quotient (IQ) were related to childhood exposure rather than exposure during pregnancy [119]. This was also supported by a recent meta-analysis, which drew the conclusion that exposure to PAEs is more dangerous during childhood than during fetal development, when it comes to IQ and psychomotor developmental index (PDI) [56]. However, research on animals, particularly in zebrafish, has shown that these compounds are able to suppress embryonic neurogenesis, a crucial process during the two life stages addressed before [57].

As for OCPs, the evidence for cognitive alterations in humans due to prenatal exposure is quite strong. Several studies have found a direct association between early exposure during pregnancy and developmental disorders such as ASD [58]. A study carried out in California showed that the risk of ASD increases after prenatal exposure to environmental pesticides within 2000 m from mother’s residence during pregnancy. For all cases of ASD, exposure during gestation was related to a 10% increase in adjusted odds ratios (ORs) for substances like glyphosate, malathion, chlorpyrifos, diazinon, permethrin, and avermectin. In addition, among cases of ASD associated with intellectual disability, ORs showed higher increases (30–40%) for many of these compounds [59]. In this sense, a gender specificity has also been reported, with a greater association between measured metabolites and ASD in girls (OR of 1.64) rather than in boys (OR of 0.84) [60]. Other studies from New York and Cincinnati cohorts support these findings, reporting an increase in ASD or augmented scores on the Social Responsiveness Scale, which is a questionnaire applied to evaluate signs of this condition. Detectable pesticide levels during gestation were related to worse internalizing (β −4.50), externalizing (β −4.74), depression (β −3.21), somatization (β −3.22), behavioral regulation (β −3.59), conduct problems (β −5.35), inhibitory control (β −7.20), and emotional control (β −3.35), among other features [120,121]. Contrarily, a study has found no association between urinary metabolites of these substances during pregnancy and ASD or attention deficit disorder [61], which is supported by Millenson et al. [121] through their examination of social skills development and risk of ASD at 8 years of age. On the other hand, negative outcomes have also been reported when it comes to child cognition and IQ scores [62,63], even though one previous longitudinal study on the subject found no association [122]. Higher results on the Child Behavior Checklist have been found after measuring in utero exposure to these pesticides [123].

OCPs are gradually being replaced by pyrethroids, but these chemicals might not be exempt from neurodevelopmental effects. In this sense, a longitudinal study showed an inverse association among exposure during pregnancy and newborn cognition [63], even though another study did not draw this conclusion [124]. This concern also exists for ASD, based on some studies carried out in areas with high use of this pesticide [59]. Furthermore, cohorts from diverse countries have reported increasing incidence of attention deficit hyperactivity disorders, with both internalizing and externalizing symptoms, associated with urinary pyrethroid levels [11].

Regarding exposure to PBDEs, it has also shown consistent negative relationships with IQ score [64,125]. Detrimental associations have been found between behavior and prenatal exposure of these chemicals [114]. South Korean research has highlighted more elevated scores on attention deficit disorder scales for children from mothers exposed to higher levels of PBDEs [65], and a study carried out in Norway even reported diverse associations for different PBDEs measured in breastmilk [66]. Studies in some other areas have failed to find similar outcomes, but this fact has been related to the lower level of exposure suffered in the counties where this research was performed, mostly Europeans ones, when compared to American ones [11].

The findings for PFASs are mixed, as some studies have failed to find a solid correlation between exposure and cognitive development at 8 years of age [67], or attention deficit disorders in preschool children [68], whereas others do report this association particularly with reduced visual motor skills. On the other hand, a study showed a correlation between exposure to PFASs during pregnancy and improved cognitive development, which represents the opposite from the hypothetically expected results [69].

Finally, prenatal nonylphenol exposure has been related to altered neurodevelopment in young children (especially boys), which has been supported by animal research reporting behavioral deficits associated with ASD in rats exposed in early life [126,127].

### 3.6. Prenatal EDCs Exposure and Psychomotor Development

**Figure 4:**
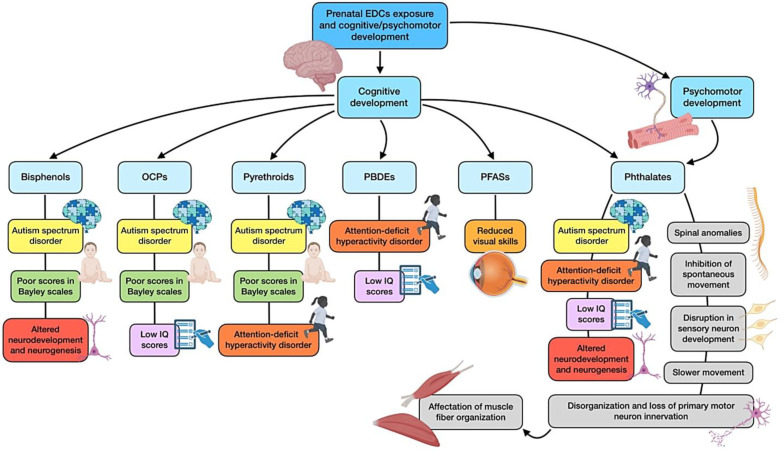
Summary of the main associations found between EDCs prenatal exposure and cognitive and psychomotor development.

Some studies have addressed psychomotor development and function in relation to EDC exposure, although research has been mainly focused on zebrafish models. Embryonic exposure of these animals to DEHP and DBP was found to significantly produce spinal anomalies, with spine curvatures and inhibited spontaneous movement. These phthalates seemed to alter the expression of genes associated with the development of the notochord, skeleton, and muscle, dampening the locomotor activity of larvae at 144 h post fertilization [70]. The most recent study investigating DBP’s involvement in motor and sensory neuron alterations supported these findings, as it showed that post fertilization administration induced disorganization and loss of primary motor neuron innervation of somatic tissue, which was accompanied by affectation of muscle fiber organization. Moreover, disruptions in sensory neuron development were also discovered, which included impairment in dorsal root ganglion and its peripheral axons, as well as loss of the bilateral soma positioning along the spinal cord and its afferent projections [71]. In other studies, psychomotor alterations have been translated to impaired behavioral patterns, including a reduction in total distance traveled and slower movement. Kim and colleagues [72] associated the impairments with inhibition to dopamine synthesis, together with an imbalance between inhibitory and excitatory neurotransmission. The effects of prenatal exposure on this matter have also been reported for several more phthalates (including DMP, DEP, DEHP, DNOP, and BBP), with spinal curvature and abnormal movement of these animals [49]. Finally, limited rodent research has provided relevant evidence on psychomotor impairments, highlighting a study in which gestational rats were exposed to DBP. This intervention resulted in a considerable incidence of skeletal alterations in fetuses, while neonates showed poor scores in sensory and motor development. In addition, some of these defects seemed to be multigenerational [128]. With regard to other EDCs, perinatal exposure to nonylphenol has been associated with delayed myelination in the newborn cerebellum, even though this situation can be recovered over time and returned to normal in adulthood [32]. Figure 4 shows a summary of the main alterations derived from prenatal exposure to EDCs on cognitive and psychomotor development.

### 3.7. Prenatal EDCs Exposure and Other Health Outcomes

Respiratory alterations: Prenatal BPA exposure has been investigated for its potential relevance in wheezing and the development of asthma [129]. Based on a prospective birth cohort, it was found that this prenatal exposure was associated with increased risk of wheeze in the offspring at 6 months, even though this relationship diminished at 3 years of age. Likewise, the association between BPA exposure and wheeze was reported at 16 weeks of gestation, but not at 26 weeks or at birth, which means a possible early critical window of exposure in pregnancy [130]. Another study examined third trimester and childhood BPA exposure regarding asthma development at 5–12 years of age. Among the main results drawn from it, exposure at 3, 5 and 7 years old correlated with asthma at 5–12 years of age, while prenatal exposure was related to reduced asthma risk at 5 years of age [131]. These contradictory results may be partially explained by the possible prenatal window of susceptibility, as neither of the mentioned studies found associations regarding late gestation exposure, while the first study found a correlation at 16 weeks. Finally, animal studies have reported asthma in mouse pups induced by prenatal BPA exposure [132].

Congenital malformations: morphology anomalies have also been a subject of study, especially regarding PAEs administration. Zebrafish embryos exposed to BBP and DBP separately, have shown several abnormalities, including yolk-sac edema, tail deformity, and un-inflated swim bladder [48,103]. Likewise, the offspring of rats exposed to DBP during gestation reported skeleton and craniofacial malformations, together with an important delay in physical growth. Additionally, these defects continue to affect the following generations of rats [128].

Eye health: Ocular alterations induced by prenatal exposure to EDCs have been addressed in animal studies. When treated with environmentally relevant concentrations of DBP, eye development defects were reported at 96h post fertilization, such as reduced lens and retina size, poor vascularization, and loss of the optic nerve and tectum [133]. On the other hand, exposure of this phthalate to pregnant rats induced anomalies in face and eye formation, the incidence of which seemed to be higher in later generations compared to F1 [128].

### 3.8. Limitations of the Study

The evaluation of EDCs’ effects is a challenging topic and becomes complicated if they are intended to be assessed in pregnant women and/or their offspring. The contrasting results obtained by several studies may be explained by the certain number of limitations that this research entails. First, there is a great deal of variation in the experimental models that may be used, with the exception of clinical studies, where the necessary manipulations are unethical to carry out on human subjects. Secondly, a variety of exposure circumstances may be assessed, taking into account the effects of combinations of compounds and their respective concentrations, distribution, and time. Several articles are just focused on a single type of chemical or its metabolites, because of the difficulty in controlling the real exposure that people experience, the cost of the performed analytical technology, as well as the substantial sample size needed to achieve statistical power results, which has restricted a simultaneous evaluation of the thousands of compounds which exert endocrine effects. The evaluation of certain EDCs with a shorter half-life contains some inaccuracies, which is another limitation to highlight. For instance, for non-persistent substances with variable concentrations, research focused on a certain location or time frame during the gestation process will probably exhibit a significant attenuation bias and less power. In this sense, collecting frequent samples across pregnancy would be a strong recommendation to reduce measurement errors. Another issue, especially for human research, is the inability to measure EDCs in target tissues, or at least, not during gestation since placentas can only be collected and evaluated after the delivery is complete. Conversely, though the use of supraphysiological doses in animal-based studies reveals immediate cytotoxic effects, the magnitudes used surpass those that humans are exposed to. This is the reason why their pharmacokinetic data and human biomonitoring should be utilized to create prediction models that enable the application of analogous dosing regimens in animals. Another limitation to consider is related to the existing gaps in our understanding of some substance’s distribution and metabolism during pregnancy, as well as the disparities in research intensity across EDCs, which makes it difficult to reach reliable judgments about poorly studied chemicals compared to those that have been more deeply assessed, like BPA or some types of phthalates.

## 4. Conclusions

Populations may come into contact with endocrine disruptors in a variety of ways, which, together with their detrimental effects on offspring development and health regarding maternal exposure, emphasize that a reevaluation of their uses and applications in the current society is required. These substances can frequently cross the placental barrier and reach the fetus, thus endangering it and increasing the risk of developmental abnormalities and NCDs in future generations. During several years of scientific research, observational studies and animal-based research has highlighted a relationship between maternal EDC exposure and detrimental repercussions in the offspring, sometimes in a sex-specific manner. However, some of these studies have reached heterogeneous results, in some cases contradictory, or do not report a relevant statistical significance in the relationship between some substances and the postnatal state of health. According to this, further research is being performed to elucidate the actual effects of maternal exposure to endocrine disruptors on the offspring. International scientific societies have been recommending the implementation of measures through specific policies, although there is still so much room for improvement regarding education of the general population, particularly pregnant mothers. Some molecules have emerged as replacements to find a solution for the demonstrated negative impact of their congeners. However, many studies point out adverse effects similar to those of the chemical they are intended to substitute. Therefore, further research is required in order to fully elucidate the detrimental effects of “traditional” EDCs, as well as the potential health damage that their substitutes may exert.

## Figures and Tables

**Table 1:** Summary of major findings in articles included and reviewed.

Reference	EDC	Study Design	Major Findings
[35]	BPA	Animal model and in vitro	Maternal exposure to BPA induces adiposity, hypertrophic adipocytes, and increased expression of pro-adipogenic and lipogenic factors in the offspring in vivo, as well as pre-adipocyte proliferation and adipocyte lipid content in vitro.
[36]	BPS	Animal model	Male mice prenatally exposed to PBS show gonadal white adipose tissue hypertrophy, with a relevant increase in adipocyte size, suggesting an augmented susceptibility to high fat diet-induced adipogenesis in male adults.
[37]	BPA	Observational	Gestational urinary BPA levels are related to subtle increases in girls’ central adiposity during childhood.
[38]	BPA	Observational	Maternal urinary BPA levels seem to be significantly associated with waist circumference in 7-year-old children, thereby increasing risk of central obesity, especially in girls.
[39]	PAEs, Bisphenols	Observational	Maternal first trimester phthalates concentration is associated with augmented pericardial fat index at 10 years old, while bisphenol concentrations do not seem to be associated with childhood adiposity. Significant sex-specific effects are not found.
[40]	PFAS	Observational	Prenatal, rather than postnatal, exposure to PFAS may contribute to a negative lipidemic profile and adiposity in childhood.
[41]	PFAS	Observational	Specific PFAS might act as developmental obesogens, with maternal race-ethnicity being relevant modifier of the associations among non-obese women.
[42]	PFAS	Observational	There are sex-specific associations with childhood adiposity regarding prenatal exposure to individual and mixed PFAS, these relationships being negative for boys and positive for girls.
[43]	PFAS	Meta-analysis	Early life PFAS exposure has an overall effect augmenting childhood overweight, also raising the z-score of childhood body mass index, which is translated to an increased risk of childhood adiposity.
[44]	PAEs	Observational	Weak negative associations are detected between maternal levels of some phthalates and height and weight z-score during infancy. Weak positive relationships between maternal concentrations of some high molecular weight PAEs and z-score are detected during childhood. Age at menarche is slightly delayed in girls with higher prenatal exposure.
[45]	PAEs (MEP), Propylparaben	Observational	Prenatal exposure to certain PAEs and parabens might augment the risk of obesity in early childhood.
[46]	PAEs	Observational	There are associations between prenatal PAEs exposure and low weight at birth, but not at childhood follow-up visits. A pattern of association with low adiposity at delivery and high adiposity at 3–4 years old is also observed.
[47]	PAEs (MBP, MIBP, MBZP)	Observational	Associations between PAEs and adiposity vary by phthalate and timing of exposure. MBP, MIBP, and MBZP in early gestation seem to be associated with adiposity among girls.
[48]	PAEs (DBP)	Animal model	DBP exposure results in developmental toxicity, pericardial edema, cardiac deformities, and changes in the expression of important cardiac transcription factors.
[49]	PAEs (DMP, DEP, DBP, DEHP, DNOP, BBP)	Animal model	All six PAEs induce different developmental abnormalities, including altered movement, reduced heartbeat, spinal curvature, and pericardial edema. DBP and BBP showed higher toxicity, as they can cause zebrafish mortality even at low doses.
[50]	BPA, PFAS	Animal model and in vitro	Combined BPA and PFAS exposure leads to morphological alterations in the fetal heart, also increasing cardiomyocyte size and collagen content.
[51]	PAEs (DEHP)	Animal model and in vitro	DEHP seems to increase apoptosis, reduce heart weight and area, slow down myocardial sarcomere development, and produce cardiac septal defect in the fetal heart.
[52]	Bisphenols (BPA, BPS, BPF)	Observational	Children’s psychomotor development is reduced across quartiles of BPS concentrations. Increases in BPA levels are related to lower mental development. Prenatal BPF exposure is not significantly related to child neurodevelopment.
[53]	BPA	Animal model	Prenatal BPA exposure alters autism spectrum disorder-related genes related to neuronal viability, neuritogenesis, and learning/memory in a sex-dependent manner, augmenting the risk of this disease in males.
[54]	PAEs	Observational	Higher prenatal PAEs levels were associated with subsequent autism spectrum disorder and adverse neurodevelopment, highlighting the importance of combined exposures.
[55]	PAEs (MIBP, MCPP, MCOP, MCNP, MEP, DEHP)	Observational	Phthalate exposure in pregnancy is not associated with autism spectrum disorder in children.
[56]	PAEs (DEHP)	Meta-analysis	There is a significant association between DEHP levels and neurodevelopmental outcomes in the offspring, as well as between DEHP exposure and psychomotor development later in childhood.
[57]	PAEs (DBP, DINP, BBP)	Animal model	Phthalates impair neurogenesis during embryonic development, partly by disrupting the expression of estrogen receptors.
[58]	OPs	Observational	There is mixed evidence linking OP exposures with developmental disorders like autism spectrum disorders. Subtle effects among populations with ubiquitous exposure are observed.
[59]	OPs, Pyrethroids	Observational	Offspring’s risk of autism spectrum disorder increases after prenatal exposure to ambient pesticides during pregnancy, with comorbid intellectual disability.
[60]	OCPs	Observational	There is an association between autism spectrum disorder and OCPs prenatal exposure among girls, as well as a lack of association in boys, so further research is needed.
[61]	OPs	Observational	No associations seem to be found for OP exposure regarding attention deficit hyperactivity disorder and autism in children.
[62]	OPs	Observational	A relationship between maternal OPs levels and low IQ scores at 6 years of age is not observed. There is some evidence for an inverse association between child nonverbal IQ and late pregnancy OPs, even though it seems to be imprecise.
[63]	Bisphenols (BPA, BPF), PAEs	Observational	Prenatal exposure to bisphenols and phthalates is associated with lower intellectual functioning at 7 years of age among boys. BPF is identified as the primary chemical of concern.
[64]	PBDEs	Observational	PBDE exposure reposts adverse effects on preschool maturity of children, having a potential negative impact on child neuropsychological development.
[65]	PAEs, BPA, PCBs, 19 OCPs, PBDEs	Observational	Maternal MEP levels are significantly associated with early mental, psychomotor, and social development. Breast milk and blood DEHP are inversely related to mental and psychomotor development. Maternal PCBs and MEP are also higher among the children with behavioral problems.
[66]	OCPs, PBDEs, PCBs, PFAS	Observational	Multi-pollutant analysis shows that early-life exposure to OCPs and PFAS is associated with a major risk of attention deficit hyperactivity disorder, with a possible sex-specific impact for PFAS. There is an unexpected inverse association between DDT and this disorder.
[67]	PFAS	Observational	Adverse associations between prenatal and childhood PFAS exposure and cognitive function at age 8 years are not observed.
[68]	PFAS	Observational	There is not consistent evidence to claim that prenatal PFAS exposure is related to attention deficit hyperactivity disorder symptoms or cognitive dysfunctions in preschool children aged 3.5 years old. There are some weak correlations between PFAS and working memory, particularly negative with nonverbal one, but also positive relationships with verbal working memory.
[69]	PFAS	Observational	There seems to be associations between prenatal/childhood PFAS exposure and low childhood visual motor abilities. On the contrary, higher prenatal PFAS reports to be related to better cognitive outcomes in some.
[70]	PAEs (DEHP, DBP)	Animal model	PAEs cause spinal birth defects, inducing transcriptional alterations of related developmental genes, which leads to altered behavior.
[71]	PAEs (DBP)	Animal model	DBP proves its toxicity to developing motor and sensory neurons during embryonic development.
[72]	BPA	Animal model	BPA developmental exposure produces behavioral alteration resulting from high accumulation and dysregulation of dopaminergic, serotonergic, cholinergic, and GABAergic neurotransmitter systems.
